# Gut microbiota as a key regulator in endometriosis: mechanisms, therapeutic opportunities, and future perspectives

**DOI:** 10.3389/fcimb.2025.1730739

**Published:** 2026-01-30

**Authors:** Xiaojun Liu, Minghui Fan, Yang Wang, Dongyun He, Li Liu

**Affiliations:** 1 Reproductive Medicine Center, China-Japan Union Hospital of Jilin University, Changchun, Jilin, China; 2 Department of Dermatology, Affiliated Hospital of Changchun University of Chinese Medicine, Changchun, Jilin, China

**Keywords:** endometriosis, estrogen metabolism, gut microbiota, inflammation, probiotics

## Abstract

Endometriosis (EMs), a common and frequently occurring gynecological disease, is a major cause of chronic pelvic pain and infertility in women. Its pathogenesis remains unclear to date, and it is characterized by high invasiveness and recurrence tendency. Although the specific pathogenesis of EMs has not been clarified, existing studies have confirmed that gut microbiota dysbiosis plays an important role in its pathogenic process. Studies suggest that gut microbiota may affect the occurrence and progression of EMs through immunoinflammatory pathways and metabolic pathways (such as enhanced estrogen metabolism and abnormal lipid metabolism). Meanwhile, approaches including dietary intervention, supplementation of probiotics or prebiotics, and microbiota transplantation can help prevent and alleviate EMs symptoms, providing potential therapeutic methods. This article will review the research progress on the correlation between gut microbiota dysbiosis and EMs, with the aim of offering more references for the diagnosis and treatment of EMs.

## 1 Introduction

Endometriosis (EMs) is a type of chronic inflammatory and hormone-dependent gynecological disease that affects the reproductive health of approximately 190 million women worldwide (Garcia et al., 2023; Czubak et al., 2025). EMs leads to changes in the pelvic microenvironment, with pain and infertility as its main manifestations (Ghosh et al., 2020; Gruber and Mechsner, 2021). The complex pathogenesis of EMs results in the lack of effective treatment options (Czubak et al., 2025). In recent years, with the explosive growth of knowledge regarding the human microbiome, a growing number of clinical trials and animal experiments have confirmed that there are significant differences in the microbiota of the intestinal tract, reproductive tract, and peritoneal cavity between EMs patients (and EMs animal models) and healthy control groups (Wei et al., 2020; Wang and Wang, 2025). Although the causal relationship between these microbiota changes and EMs has not yet been clarified, current research evidence supports the existence of a bidirectional relationship between the two (Cao et al., 2023; Li et al., 2025). A growing body of evidence underscores gut microbiota dysbiosis as a critical, yet underexplored, driver of EMs (Iavarone et al., 2023), and this review offers three distinct strengths to advance the field: first, it synthesizes cutting-edge evidence to clarify the causal link between gut microbiota imbalance and EMs progression, going beyond correlative observations to delineate core mechanisms—including immunoinflammatory dysregulation, enhanced estrogen bioavailability, and aberrant lipid metabolism—that directly modulate ectopic lesion growth and tissue invasion; second, it provides a systematic, evidence-based overview of translatable therapeutic strategies (dietary intervention, probiotic/prebiotic supplementation, and fecal microbiota transplantation), highlighting their potential to target gut microbiota dysbiosis and alleviate EMs symptoms, a critical gap in current treatment paradigms that rely heavily on hormonal therapy or surgery; third, it integrates emerging multi-omics data (microbiomics, metabolomics, and transcriptomics) to identify novel diagnostic biomarkers, addressing the unmet need for non-invasive early detection of EMs. By consolidating the latest research on gut microbiota-EMs crosstalk, this review not only strengthens the rationale for targeting the gut microbiome in EMs management but also outlines future directions for personalized therapeutic development, offering valuable references for clinicians and researchers alike.

## 2 Characteristic alterations in the gut microbiota of EMs

### 2.1 Dysbiosis of the gut microbiota in patients with EMs

A complex and diverse community of microorganisms inhabits the human gut. These gut microbial communities not only play a crucial role in maintaining host health but are also closely associated with the occurrence and development of various diseases, including EMs (Ha et al., 2014; Ni et al., 2020). As the largest microbial community in the human body, the gut microbiota is mainly composed of two dominant phyla: Bacteroidetes and Firmicutes (Mahowald et al., 2009). Together, these two phyla account for approximately 95% of the gut microbiota in healthy populations. An increased Firmicutes/Bacteroidetes (F/B) ratio is widely recognized as a key characteristic of gut dysbiosis (Stojanov et al., 2020; Yin et al., 2025). Multiple studies have shown that this ratio is elevated in patients with EMs, indicating that gut dysbiosis is prevalent among EMs patients (Wang et al., 2025). A systematic review on the microbial characteristics of EMs (Leonardi et al., 2020) found that at the phylum level, the abundances of Actinobacteria, Firmicutes, Proteobacteria, and Verrucomicrobia were significantly increased in the gut, while the abundance of Bacteroidetes was significantly decreased. These findings suggest that EM is associated with increased counts of various microbial taxa, including Proteobacteria, Enterobacteriaceae, Streptococcus, and Escherichia coli. Shan et al (Shan et al., 2021), by analyzing fecal samples from patients with ovarian endometriosis (OE) and deep infiltrating endometriosis (DIE), found that compared with healthy individuals, the abundance of Firmicutes and Clostridia in the gut of OE and DIE patients was significantly decreased, while the abundance of specific genera such as Ruminococcaceae was significantly increased. Similar conclusions were also drawn from the studies by Svensson (Svensson et al., 2021).

**Figure 1:**
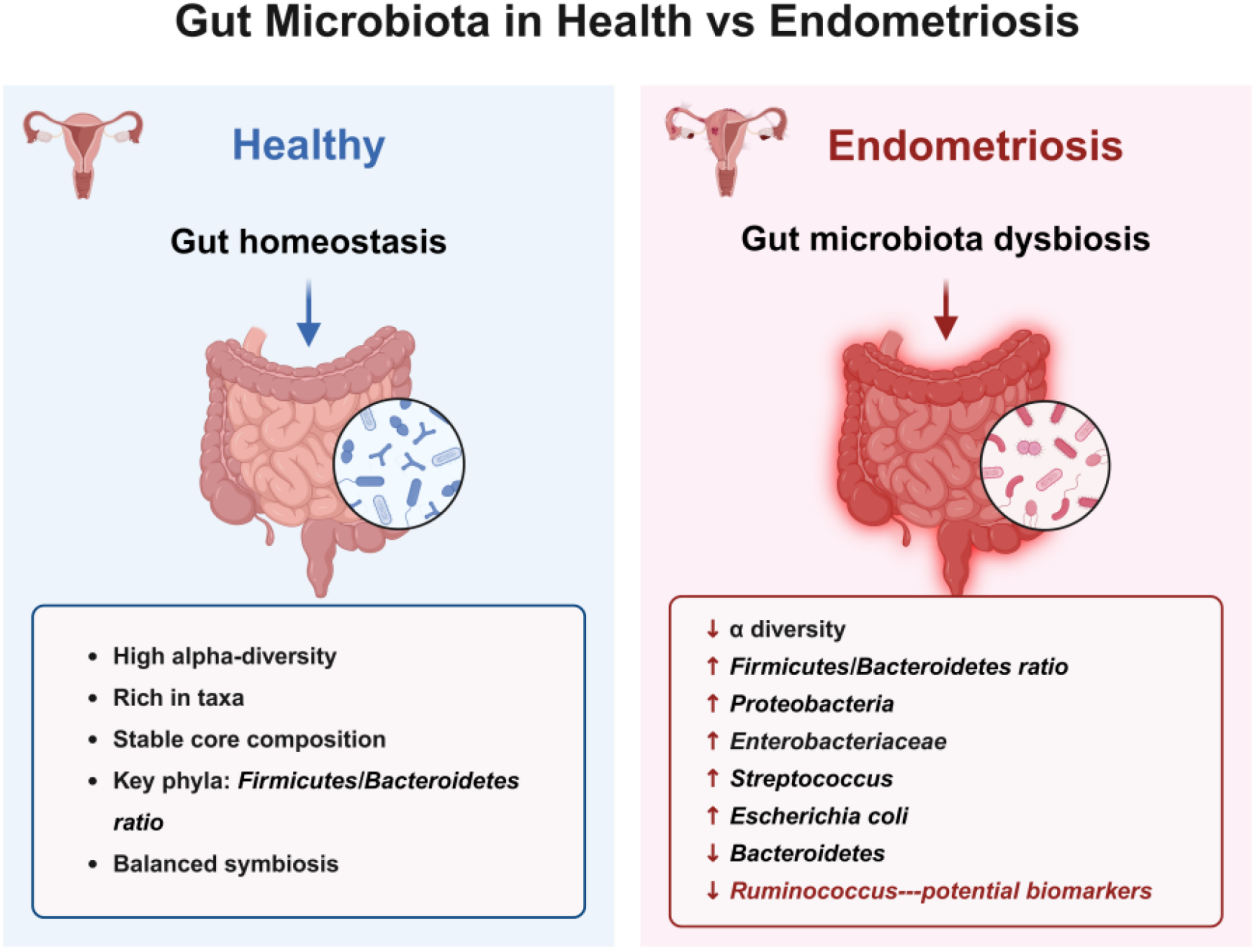
Gut microbiota in healthy women and EMs patients.

Alterations in the gut microbiota may play a key role in the pathogenesis of EMs. In particular, certain specific genera of the gut microbiota, such as *Ruminococcus*, have been identified as taxa with high diagnostic value through robust machine learning methods (Huang et al., 2021). As potential biomarkers, gut microbiota has shown more superior performance in the non-invasive diagnosis of EMs compared to cervical and peritoneal microbiota (Huang et al., 2021). Shan et al (Leonardi et al., 2020). performed 16S rRNA gene sequencing on the gut microbiota in fecal samples from 12 patients with stage III-IV EMs and 12 healthy controls. The results showed that compared with the control group, the EMs group had lower alpha diversity of gut microbiota and a higher Firmicutes/Bacteroidetes ratio. Additionally, there were significant differences in the abundance of microbiota such as *Actinobacteria*, *Tenericutes*, *Blautia*, *Bifidobacterium*, *Dorea*, and *Streptococcus* between the two groups. These studies confirm that there are significant differences in the composition ratio of gut microbiota between EMs patients and healthy women (**Figure 1**).

### 2.2 Dysbiosis of the gut microbiota in EMs animal models

To further explore the causal relationship between gut microbiota and EMs, researchers have established EMs animal models. Yuan et al (Yuan et al., 2018). induced the establishment of an EMs mouse model via intraperitoneal injection of endometrial tissue and monitored the composition of gut microbiota. Forty-two days after model establishment, they found significant changes in the gut microbiota in the feces of EMs mice: increased abundance of Firmicutes and Actinobacteria, and decreased abundance of Bacteroidetes, indicating that EM induces gut microbiota dysbiosis. Chadchan (Chadchan et al., 2019; Chadchan et al., 2023) found that after treatment with broad-spectrum antibiotics or metronidazole, mice with gut microbiota depletion showed a significant reduction in EMs lesions and alleviated inflammation. Furthermore, when these mice were given oral gavage of feces from EMs mice, the EMs lesions and inflammation in their bodies were restored. Interestingly, when the same mice were given oral gavage of feces from healthy mice, no restoration of EMs lesions was observed. These two important studies demonstrate the interaction between EMs and gut microbiota: the microorganisms in the feces of EMs mice can induce EMs and inflammation, and the development of EMs requires the involvement of gut microbiota. In another mouse experiment, 6 EMs mice were intervened with the Chinese herbal medicine Shaofu Zhuyu Decoction for 21 days, and 16S rRNA gene sequencing analysis was performed on their fecal samples (Cao et al., 2020). The results showed that this Chinese herbal medicine could regulate carbohydrate, amino acid, and lipid metabolism in the mouse gut, increase the diversity of gut microbiota, and significantly reduce the abundance of *Lachnospiraceae* (P<0.05), *Rikenellaceae* (P<0.01), *Ruminococcaceae* (P<0.01), *Lachnoclostridium* (P<0.05), and *Candidatus Ruminococcaceae* (P<0.05), thereby effectively reducing the volume of ectopic lesions and the degree of fibrosis. Subsequent pairwise comparisons revealed that between the non-EMs group and the EMs group, as well as between the EMs group and the EMs group treated with the Chinese herbal medicine, significant statistical differences were observed in *Rikenellaceae* (P<0.01), *Oscillospiraceae* (P<0.05), *Tannerellaceae* (P<0.05), *Alistipes* (P<0.05), *Candidatus Oscillospiraceae* (P<0.05), and *Rikenella* (P<0.05) (Cao et al., 2020). These studies mentioned above indicate that specific microbiota are closely associated with the occurrence of EMs and may play a role in its pathogenesis.

## 3 Mechanisms underlying gut microbiota-mediated EMs pathogenesis

EMs is an estrogen dependent chronic inflammatory disease, and immune inflammatory response is the pathological basis. The microbiota may participate in the pathological mechanism of EMs through various pathways such as mediating inflammatory response, regulating immune response, participating in estrogen regulation, and interfering with metabolic activity.

### 3.1 Imbalance of gut microbiota affects the release of inflammatory factors

The occurrence and progression of EMs are closely associated with inflammation, and the activation of inflammatory pathways is involved in the pathogenesis of EMs, with bacteria and their metabolites participating in this process (Tang et al., 2024).

LPS is a key component of the outer membrane of Gram-negative bacteria, and it normally exists in sites such as the skin, oral cavity, and gastrointestinal tract (Sperandeo et al., 2017; Giordano et al., 2020). Elevated LPS levels induce the massive growth and reproduction of intestinal pathogenic bacteria while inhibiting the activity of beneficial bacteria (Canny and McCormick, 2008). When the body’s immunity declines or it is infected, increased LPS content in the blood stimulates endometriotic stromal cells to produce large amounts of tumor necrosis factor-α (TNF-α) and interleukin-8 (IL-8). This also enhances the mitotic activity of human endometriotic stromal cells, while upregulating the expression of cyclooxygenase-2 (COX-2) and prostaglandin E2 (PGE2), thereby promoting the proliferation and invasion of human endometriotic stromal cells (Iba et al., 2004). During menstruation in women, the intravaginal environment changes, and gut microbiota can enter the abdominal cavity through retrograde menstruation (Shen et al., 2022). This leads to a significant increase in LPS levels in the peritoneal fluid of EMs patients, inducing the production of inflammatory factors and angiogenesis factors in the abdominal cavity, and enabling the implanted growth of retrograded endometrial fragments in the abdominal cavity to form ectopic lesions (Chen et al., 2023). The inflammatory response mediated by the gut microbiota LPS-TLR4 pathway is mainly dominated by Proteobacteria. Moreover, patients with gut microbiota dysbiosis exhibit increased TLR4 expression in peripheral blood monocytes and elevated inflammatory levels in peripheral blood (Yang et al., 2020; Candelli et al., 2021). When gut microbiota dysbiosis occurs, the proportions of tight junction proteins and occludin between the host’s intestinal epithelial cells decrease, resulting in increased intestinal mucosal permeability (Allam-Ndoul et al., 2020; Stolfi et al., 2022). LPS produced by gut microbiota metabolism enters the blood and binds to lipopolysaccharide binding protein (LBP), activating the inflammatory cascade reaction in the body (Ribatti, 2024). This places the body in a state of low-grade inflammation, which induces the development of EMs (Bausic et al., 2025). In summary, the occurrence and progression of EMs are closely related to inflammation, and the activation of inflammatory pathways is involved in the pathogenesis of EMs, with bacteria and their metabolites involved.

### 3.2 Intestinal microbiota participates in immune system regulation in patients with EMs

The pathological changes of endometriosis (EMs) are characterized by the migration of endometrial glands and stroma with growth function to sites outside the uterine body, where they form ectopic lesions (Laganà et al., 2019; Hosseinirad et al., 2025). These ectopic lesions undergo periodic proliferation and bleeding under the influence of estrogen, and such pathological changes induce the activation of the immune system in the peritoneal cavity. Short-chain fatty acids (SCFAs) produced by the colonic fermentation of resistant starch are considered to be involved in autoimmune regulation (Topping and Clifton, 2001). After SCFAs bind to their G protein-coupled receptors (GPCRs), they exert immunomodulatory effects (Ratajczak et al., 2019). Knockout of the G protein-coupled receptor kinase-interacting protein 2 (GIT2) gene causes changes in the gut microbiota of mice, and compared with wild-type mice, the degree of intestinal damage in these gene-knockout mice is significantly reduced (Le et al., 2024).

In the gut microbiota, Firmicutes (specifically *Clostridium butyricum*) can metabolize to produce butyrate, while *Bifidobacterium* and Bacteroidetes generate acetate (Duncan et al., 2004; Fu et al., 2019). Butyrate promotes the differentiation of naive T cells into regulatory T cells (Treg cells) and directly regulates T cell responses (Furusawa et al., 2014; Takahashi et al., 2020). Acetate and butyrate can modulate the interaction of dendritic cell-T cell complexes (DC-T complexes) (Zeng and Chi, 2015). By acting as histone deacetylase inhibitors (HDACi), they inhibit the expression of nuclear factor κB (NF-κB) and induce the transcriptional activation of anti-inflammatory genes to activate dendritic cells, thereby promoting the differentiation of Treg cells and maintaining immune homeostasis (Park et al., 2015). Acetate inhibits the maturation and metabolism of LPS-induced human monocyte-derived dendritic cells, promotes the polarization of naive CD4+ T cells into Treg cells that secrete interleukin-10 (IL-10), and increases the production of colonic immunoglobulin A (IgA) (Wang et al., 2023; Shvets et al., 2024). This enhances the binding capacity of IgA to gut microbiota and strengthens the protective function of the intestinal mucosal immune barrier (Pietrzak et al., 2020). Gut microbiota-derived butyrate could inhibit endometriotic lesions and human endometriotic cell survival through regulating histone deacetylases and G-protein-coupled receptors (Chadchan et al., 2021). Gut microbiota-derived acetate exhibits anti-EMs role through regulating M1 macrophage polarization by activating JAK1/STAT3 pathway (Xu et al., 2025).

Metabolites of the gut microbiota and the endotoxins they produce weaken the intestinal barrier (Gasaly et al., 2021). Increased intestinal mucosal permeability leads to the “leaky gut” phenomenon, allowing various inflammatory factors and toxic substances to enter the bloodstream (Macura et al., 2024). This triggers various antigen-antibody bindings and induces immune responses (Escalante et al., 2025). Interleukin-37 (IL-37) is a natural anti-inflammatory cytokine that participates in gut microbiota regulation and immune responses (Xu et al., 2015; Su and Tao, 2021). Gut microbiota dysbiosis increases the expression of IL-37, which recruits neutrophils and natural killer cells in the colonic lamina propria and mesenteric lymph nodes. This causes damage to the intestinal epithelial barrier and enhances inflammatory responses and immune dysregulation (Cong et al., 2022; Wang et al., 2024). A previous study demonstrated that recombinant human IL-37 suppress EMs development through inducing DC cell maturation (Li et al., 2021). Another study showed that IL-37 inhibited EMs development by regulating the biological behavior of endometrial stromal cells (Jiang et al., 2018). T helper cell 17 (Th17) and regulatory T cells (Treg cells) are key subsets of CD4+ T cells (González-Amaro and Marazuela, 2016). They can secrete pro-inflammatory cytokines such as IL-17, IL-22, and IL-23, and play a crucial role in maintaining the body’s normal immune function (Noack and Miossec, 2014). Segmented filamentous bacteria (SFB) and *Clostridium leptum* participate in the differentiation of Th17 cells and promote the induction, migration, and proliferation of Treg cells (Schnupf et al., 2017; Wang et al., 2019). *Bacteroides thetaiotaomicron* is one of the most abundant bacteria in the human gut (Zocco et al., 2007). It can regulate the activity of adenosine monophosphate (AMP) in intestinal mucosal cells via Toll-like receptors (TLRs), thereby influencing the differentiation of naive T cells (Yu and Huang, 2013). Metabolites of the gut microbiota can regulate innate lymphoid cells (ILCs) (Guo et al., 2023). ILC3 is involved in the regulation of intestinal immunity, inflammation, and intestinal tissue homeostasis (Domingues and Hepworth, 2020). The proportion of ILC2 and ILC3 was decreased in patients with EMs (Sugahara et al., 2022). The lymphotoxin-α3 (LT-α3) released by ILC3 promotes the secretion of mucosa-associated IgA, participates in maintaining gut microbiota homeostasis, resists the overproliferation of pathogenic bacteria, and inhibits autoimmune responses (Liu et al., 2020). When the proportion of gut microbiota is imbalanced and the number of pathogenic bacteria increases, TLR5 in intestinal mucosal epithelial cells and dendritic cells is activated (Shi et al., 2017; Feng et al., 2023). This induces immune responses in T cells and B cells, leading to intestinal immune-inflammatory responses (Li et al., 2018). The mutual crosstalk and functional interactions among immune cells, endometrial cells, endothelial cells, and cytokines represent the most critical drivers of pathological alterations in endometriosis—including aberrant invasion, uncontrolled proliferation, and pathological adhesion of endometrial tissue. Elucidating how gut microbial dysbiosis mediates immune dysregulation thereby emerges as a pivotal direction for future research in this field.

### 3.3 The gut microbiota is involved in the regulation of sex hormones

The gut microbiota is involved in the metabolism and circulation processes of sex hormones (Yoon and Kim, 2021; Santos-Marcos et al., 2023). Based on the relationship between them, a new concept-”microgenderome” has been proposed, which refers to the interplay among gender, sex hormones, and the gut microbiome (Aguilera et al., 2020). This concept primarily illustrates the mutual interaction between sex hormones and the gut microbiome.

It has been confirmed that estrogens and androgens can directly affect the gut microbiome and immune cells (Pace and Watnick, 2021). EMs is an estrogen-dependent disease, closely associated with estrogen levels (Kitawaki et al., 2002). The gut microbiota serves as a key regulator of circulating estrogen, and it participates in the regulation of estrogen levels via β-glucuronidase (Sui et al., 2021). Hu et al (Hu et al., 2023). found that the gastrointestinal bacterial community in EMs patients exhibits higher diversity and abundance, with increased secretion of β-glucuronidase. This enzyme decouples estrogen into its active metabolites, thereby promoting estrogen downstream effects. Another study found that increased β-glucuronidase expression in EMs lesions compared to normal endometrium. And β-glucuronidase promoted EMs development directly or indirectly by causing macrophage dysfunction (Wei et al., 2023). A study conducted estrogen metabolism analysis on EMs patients. It used liquid chromatography/tandem mass spectrometry (LC-MS/MS) to quantify urinary estrogens and next-generation sequencing (NGS) technology to evaluate the microbiome data of the V4 region of 16S rRNA. The results showed that compared with healthy individuals, EMs patients had significant differences in the levels of 17β-estradiol, 16-keto-17β-estradiol, 2-hydroxyestradiol, and 2-hydroxyestrone. Additionally, the gut microbiota of EMs patients showed a significant positive correlation with urinary estrogens (Le et al., 2021). Another study showed that increased 4-hydroxyestrone (4OHE1), 2-hydroxyestradiol (2OHE2), and 4-hydroxyestradiol (4OHE2) were observed in eutopic endometrium of EMs patients (Othman et al., 2021). Shan et al (Shan et al., 2021). observed that the serum estradiol (E2) level in EMs patients is significantly elevated, and it shows a positive correlation with the abundance of Blautia and Dorea genera in feces. Meanwhile, the gut microbiota plays an important role in the reabsorption of active hormones through enzymatic and other pathways. Studies have reported that certain gut microbiota, such as Ruminococcaceae and Clostridia, affect serum estrogen levels. However, the relationship between gut microbiota and estrogen levels may be bidirectional (Yuanyue et al., 2025). On one hand, sex hormones (e.g., estrogen, progesterone) directly modulate gut microbial diversity and function by regulating gut epithelial barrier integrity, mucus secretion, and microbial metabolic pathways. On the other hand, gut microbial dysbiosis reciprocally alters systemic hormone homeostasis—for instance, via microbial enzymes (e.g., β-glucuronidase) that enhance estrogen bioavailability or modulate steroid hormone metabolism, thereby amplifying the pro-inflammatory and pro-invasive milieu driving ectopic lesion formation (Kumari et al., 2024; Tang et al., 2025). The gut microbiota may be involved in the development and clinical symptoms of EMs by influencing estrogen levels, while EMs and its associated hormone levels may also affect the composition of the gut microbiota.

More and more evidence supports the involvement of gut microbiota and estrogen in regulating reproductive, neurological, and metabolic homeostasis (Qi et al., 2021). According to reports, β-glucuronidase and β-glucosidase produced by Bacteroides, Bifidobacterium, Escherichia coli, and Lactobacillus in the intestine can promote the breakdown of estrogen, increase the reabsorption of free estrogen, and lead to high levels of circulating estrogen (Weber et al., 2024). Estradiol is essential for the attachment, implantation, survival, and production of inflammatory substances such as metalloproteinases, cytokines, prostaglandins, and growth factors in endometrial tissue. Increasing estrogen levels can stimulate the growth and inflammatory activity of endometriosis lesions. Therefore, high estrogen exposure caused by gut microbiota may be a risk factor for the occurrence and development of EMs (Baker et al., 2017).

## 4 Targeting gut microbiota for the prevention and treatment of EMs

### 4.1 Diet

**Figure 2:**
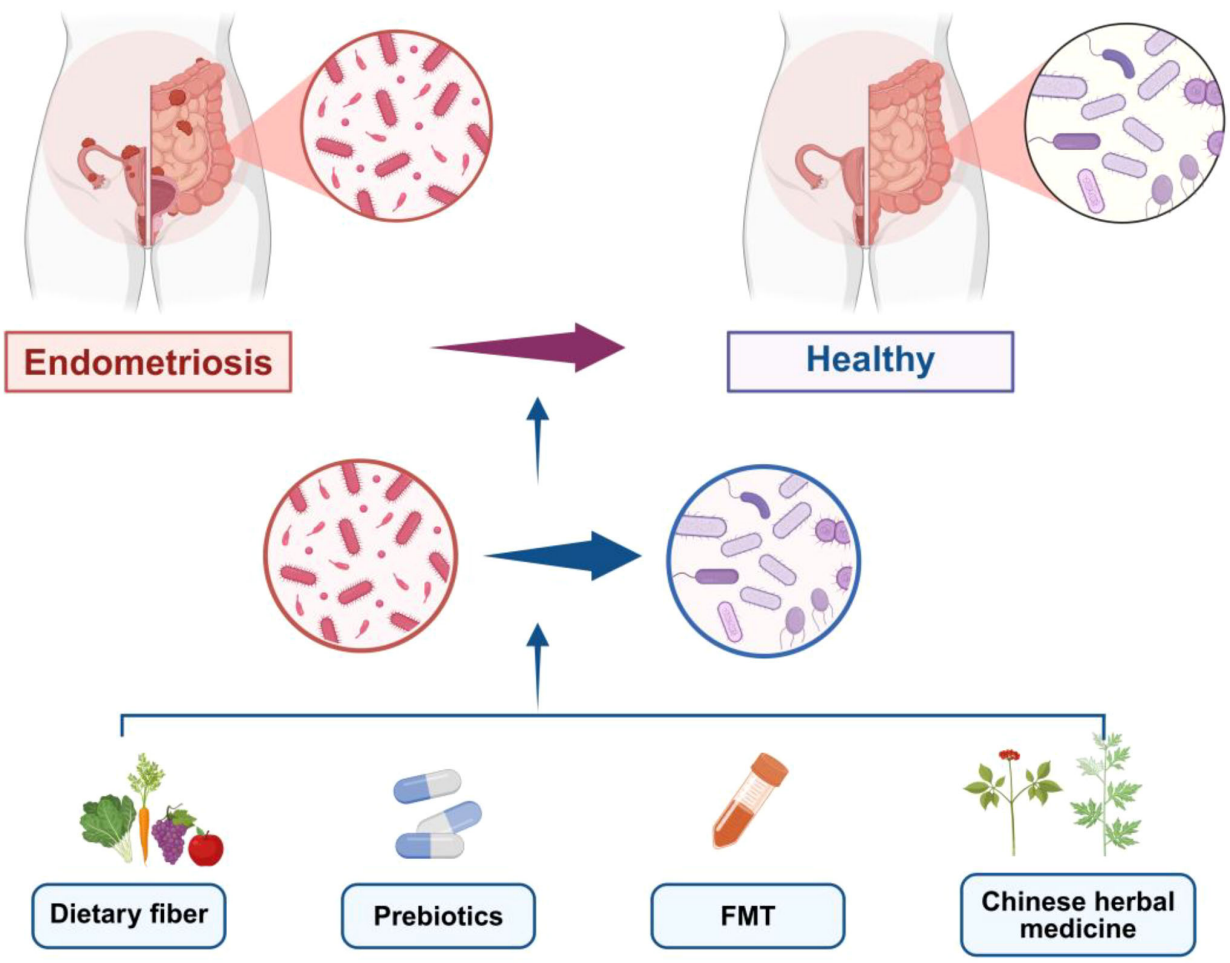
Targeting gut microbiota for the prevention and treatment of EMs.

The correlation between diet and the pathogenesis of EMs has been confirmed (Parazzini et al., 2013; Zhang et al., 2025). Women who consume large amounts of fruits, vegetables, dairy products, as well as fish and nuts rich in polyunsaturated fatty acids (PUFAs) (e.g., Omega-3 fatty acids, Omega-6 fatty acids) have a reduced risk of developing EMs (Abodi et al., 2022). Whereas consuming relatively large amounts of trans fat-rich products, red meat, and alcohol increases the risk of EMs (Missmer et al., 2010). A Western diet significantly exacerbates lesion size in a mouse model of EMs. Western diet induces depletion of *Akkermansia muciniphila* in intestinal microbiota, an important producing bacterium (Parpex et al., 2024). Omega-3 PUFAs are found in fish and nuts. Fish oil has been proven to reduce the level of prostaglandins in the circulation and alleviate dysmenorrhea and inflammation (Yamamoto et al., 2018). In EMs animal models, it has also been confirmed that diets with high Omega-3 PUFA intake exert anti-inflammatory effects and inhibit the growth of EMs lesions (Tomio et al., 2013). Omega-3 PUFAs can reduce inflammation through multiple pathways: regulating the composition of gut microbiota (e.g., increasing the abundance of beneficial bacteria such as *Bifidobacterium* and *Lactobacillus* in the distal intestine) to improve its metabolic activity, reducing inflammation-inducing precursors, regulating the expression of inflammation-related genes, and mediating the activation of immune cells via the nuclear factor κB (NF-κB) and mitogen-activated protein kinase (MAPK) signaling pathways (Fu et al., 2021). In addition, adjusting dietary patterns, such as adopting a low-FODMAP (fermentable oligosaccharides, disaccharides, monosaccharides, and polyols) diet-is also beneficial for alleviating EM-related symptoms. The therapeutic effect of the low-FODMAP diet was associated with changes in the fecal microbiota and the fecal fermentation index. The low-FODMAP diet intervention resulted in the depletion of carbohydrate-fermenting bacterial genera (e.g., *Bifidobacterium*, *Bacteroides*) and a concomitant decline in saccharolytic fermentation activity, with such changes exhibiting a positive association with symptomatic improvement among responders (Zhang et al., 2021). It is worth noting, however, that the efficacy of diet-based interventions for EMs is subject to notable interindividual variability, which stems primarily from baseline differences in gut microbiota composition and host physiological characteristics. Distinct microbial signatures shaped by genetics, lifelong dietary habits, geographic origins, and comorbidities determine how the gut microbiome responds to identical dietary adjustments. This heterogeneity underscores the challenge of developing one-size-fits-all dietary guidelines for EMs and highlights the need for personalized dietary strategies tailored to an individual’s unique microbiota profile in future translational research (**Figure 2**).

### 4.2 Probiotics and prebiotics

Reducing harmful bacteria and increasing beneficial bacteria is the most direct method to regulate gut microbiota balance. Probiotics are viable microorganisms that help restore microbial balance in the gut to improve gut health. They maintain immune system homeostasis by promoting anti-inflammatory responses and regulating immune cell activity (Markowiak and Slizewska, 2017), thereby reducing the formation of EM-related inflammatory environments. An animal experiment (Peter et al., 2018) reported that after intrauterine administration of Lactobacillus, the intrauterine inflammatory environment of dairy cows with endometritis was controlled, and their fertility was improved. Iton et al (Itoh et al., 2011a; Itoh et al., 2011b). designed a randomized, double-blind, placebo-controlled study and found that oral administration of Lactobacillus gasseri OLL2809 could significantly alleviate dysmenorrhea in EMs patients. Subsequent further animal experiments revealed that oral administration of Lactobacillus gasseri OLL2809 could effectively inhibit the growth of EM lesions by increasing interleukin-12 (IL-12) concentration and enhancing the cytotoxic activity of natural killer (NK) cells in the peritoneal cavity of mice. Another study also reported that oral administration of Lactobacillus is beneficial for reducing the severity of dysmenorrhea, dyspareunia, and chronic pelvic pain in EMs patients. It is critical to emphasize, however, that the therapeutic efficacy of probiotics is strictly strain-specific rather than universally applicable across Lactobacillus species or other probiotic genera. Not all Lactobacillus strains confer equivalent benefits. Prebiotics refer to compounds that cannot be digested by the host but can be utilized and fermented by probiotics. To improve the effectiveness of probiotics, increasing prebiotics can selectively promote the proliferation and metabolism of probiotics, thereby shaping the composition and function of gut microbiota beneficial to host health (He and Shi, 2017). While the role of probiotics in EMs has gained preliminary attention, it is important to acknowledge that direct research investigating the relationship between prebiotics and EMs remains extremely limited. Most insights into the potential relevance of prebiotics to EMs are derived from their well-documented effects on gut microbiota modulation, inflammation regulation, and immune homeostasis—pathophysiological processes closely intertwined with EMs development and progression. Despite current limitations, the established role of prebiotics in inflammation and microbiota regulation positions them as a promising area for future EMs research and therapeutic development.

### 4.3 Fecal microbiota transplantation

Fecal microbiota transplantation (FMT), a therapeutic approach to restore the normal function of gut microbiota, involves isolating microbially manipulated communities from the feces of healthy donors and then infusing them into the patient’s gut (Biazzo and Deidda, 2022; Quaranta et al., 2022). Currently, the therapeutic efficacy of FMT in the treatment of Clostridium difficile infection (CDI) has been clearly confirmed and gained broad expert consensus (Cammarota et al., 2019). Its main mechanisms include competing with the indigenous microbiota, restoring the metabolism of SBA (secondary bile acids) in the gut, and repairing the intestinal barrier by stimulating the intestinal mucosal immune system (Khoruts and Sadowsky, 2016). FMT also holds potential as a tool for the future treatment of female reproductive tract diseases (Quaranta et al., 2019). In the context of EMs, Chadchan et al (Chadchan et al., 2021). found that after treatment with FMT from healthy mice, the number of lesions in EMs mice decreased. Ni et al (Ni et al., 2021). also reported similar findings. A recent study using a mouse model of EMs reported that FMT from healthy donors altered the recipient mice’s gut microbiota composition. Mechanistic analyses indicated that FMT from healthy donors alleviated EMs via a cascade of microbiota-driven effects. It first remodeled the gut microbiota by increasing α-diversity, augmenting Lactobacillus levels, and reducing Bacteroidetes. This restructuring led to a significant rise in acetate content in both feces and ectopic lesions, which subsequently triggered the activation of the JAK1/STAT3 pathway within lesion tissues, driving the polarization of macrophages toward the M1 phenotype (Xu et al., 2025).

Vaginal microbiota transplantation (VMT), a new field in microbiota transplantation, is also emerging. Exploratory studies (Lev-Sagie et al., 2019) have reported positive results regarding VMT as an alternative treatment for refractory bacterial vaginosis (BV). Given the ascending infection pathway of pathogenic microorganisms through the vagina and cervix, VMT may also become a long-term management approach for EMs in the future. Lu et al (Lu et al., 2022). treated EM-bearing mice with VMT and gonadotropin-releasing hormone agonist (GnRH-a). Comparative results showed that both treatments exerted almost identical effects in inhibiting the growth of EMs lesions. Meanwhile, the levels of inflammatory cytokines decreased, and key proteins in the NF-κB signaling pathway were downregulated. These findings indicate that vaginal microbiota can promote the development of EMs, while VMT exerts beneficial effects on EMs.

Microbiota transplantation is a highly promising approach. However, the standardization of microbial isolation, formulation, dosage, and administration timing-aimed at ensuring optimal microbiota transplantation and maintenance to generate effective clinical responses-are all practical challenges that need to be addressed. Meanwhile, although there are significant parallels between VMT and FMT, notable physiological and clinical differences must be considered when developing treatment protocols, establishing regulatory frameworks, and evaluating therapeutic efficacy. For instance, compared with the treatment of Clostridium difficile infection (CDI), the treatment of bacterial vaginosis (BV) requires greater consideration of the characteristics of pathogenic bacteria (which are non-single opportunistic pathogens) and the issue of preventing recurrence (DeLong et al., 2019). Therefore, more thorough research and testing will be needed in the future to ensure the efficacy and feasibility of microbiota transplantation technology for different diseases. It is crucial to emphasize, however, that all EMs-related evidence supporting FMT and VMT currently derives exclusively from preclinical animal studies; no clinical data validate their efficacy or safety in human EMs patients, and no implications of established therapeutic benefit should be inferred.

### 4.4 Herbal medicine

Herbal medicine, particularly traditional Chinese medicine (TCM) formulations, has emerged as a potential complementary approach for endometriosis (EMs) intervention, with accumulating evidence supporting its efficacy in alleviating symptoms and modulating pathological processes (Ilhan et al., 2019). A representative TCM formula, *Shaofu Zhuyu Decoction*, has been extensively studied in EMs models (Zhu et al., 2018). In a mouse experiment, 21-day intervention with *Shaofu Zhuyu Decoction* significantly reduced the volume of ectopic lesions and fibrosis in EMs mice. Mechanistically, this formula exerted regulatory effects on gut microbiota composition-specifically decreasing the abundance of pro-inflammatory taxa such as *Lachnospiraceae*, *Rikenellaceae*, and *Ruminococcaceae*, while improving gut metabolic functions (e.g., carbohydrate, amino acid, and lipid metabolism). These changes collectively contributed to mitigating the inflammatory microenvironment associated with EMs.

Beyond TCM formulations, single herbal components have also shown therapeutic potential. For example, compounds derived from *Curcuma longa* (curcumin) and *Glycyrrhiza uralensis* (glycyrrhizin) have been reported to inhibit the proliferation of endometriotic stromal cells by downregulating estrogen receptor expression and suppressing the NF-κB inflammatory signaling pathway-key pathways driving EMs progression (Zhang et al., 2013; Wang et al., 2017). A recent study demonstrated that Alpha-linolenic acid (ALA) could inhibit EMs development through regulating gut microbiota, maintaining intestinal barrier, and suppressing LPS production. ALA significantly increased the abundance of *Lactobacillus*, *Bacteroides*, *Muribaculum*, *Clostridium_ sensu_ stricto_ 1* and *Bifidobacterium.* Among the genera linked to EMs research, *Bacteroides, Bifidobacterium*, and *Muribaculum* exert distinct effects on SCFA production and EMs-related pathophysiological processes. These bacterial taxa are capable of generating short-chain fatty acids (SCFAs), which in turn suppress inflammatory responses and exert therapeutic potential in the management of EMs. Notably, the efficacy of herbal medicine in EMs often involves multi-target regulation: it not only targets local ectopic lesions but also modulates systemic factors such as gut microbiota homeostasis, immune responses, and hormone metabolism-aligning with the complex pathological nature of EMs. However, current research still faces challenges, including the lack of standardized herbal extracts, inconsistent dosage regimens, and limited large-scale clinical trials. Future studies need to focus on optimizing herbal formulations, clarifying precise molecular mechanisms, and verifying clinical efficacy through rigorous randomized controlled trials to promote the rational application of herbal medicine in EMs treatment (**Figure 2**).

## 5 Conclusion

EMs is a common gynecological disease, and its incidence has been on the rise year by year. The pain and infertility caused by EMs pose a severe threat to women’s physical and mental health. The multi-factorial relationship established between gut microbiota and EMs also plays an important role in the occurrence and development of EMs. With the development of molecular omics and the popularization of high-throughput sequencing technology, research on the correlation between microbiota and the pathogenesis of EMs will become increasingly in-depth. Although there are many current studies on the relationship between gut microbiota and EMs, certain limitations still exist: (1) Most studies on the relationship between gut microbiota and EMs focus on the correlation level, while studies on their specific mechanisms of action remain insufficient; (2) There are individual differences in the quantity and species of human gut microbiota, and its individualized application is a key issue that requires further research and exploration in the future. In the future, in-depth studies on the correlation between gut microbiota and EMs should be conducted from the perspectives of systemic immunity, metabolism, and oncology. Additionally, starting from the perspectives of hypoxic microenvironment and stem cells, gut microbiota should be regulated through approaches such as antibiotics, FMT, probiotics, and nutrients to intervene in EMs, thereby providing new ideas for the clinical treatment of EMs.
